# Nephrology: a flourishing field with plentiful emerging topics

**DOI:** 10.3389/fmed.2024.1463540

**Published:** 2024-08-02

**Authors:** Kyung Don Yoo, Chia-Ter Chao

**Affiliations:** ^1^Division of Nephrology, Department of Internal Medicine, Ulsan University Hospital, University of Ulsan College of Medicine, Ulsan, Republic of Korea; ^2^Basic-Clinical Convergence Research Institute, University of Ulsan, Ulsan, Republic of Korea; ^3^Disaster Preparedness and Response Committee, The Korean Society of Nephrology, Seoul, Republic of Korea; ^4^The Korean Society of Geriatric Nephrology, Seoul, Republic of Korea; ^5^Department of Internal Medicine, Min-Sheng General Hospital, Taoyuan City, Taiwan; ^6^Division of Nephrology, Department of Internal Medicine, National Taiwan University Hospital and National Taiwan University College of Medicine, Taipei, Taiwan; ^7^Graduate Institute of Toxicology, National Taiwan University College of Medicine, Taipei, Taiwan; ^8^Graduate Institute of Medical Education and Bioethics, National Taiwan University College of Medicine, Taipei, Taiwan

**Keywords:** artificial intelligence, chronic kidney disease, clinical trial, glucagon-like peptide-1 receptor agonist, IgA nephropathy, nephrology education, sodium-glucose cotransporter inhibitors, patient reported outcome measure

The word “nephrology”, the discipline that focuses on the biology and medical implications of studies surrounding the kidney, was first coined in 1960s based on the French word “néphrologie” and the Greek word “nephrós”. Our understandings of kidney biology evolve successively over time, from the elucidation of glomeruli, nephrons, and tubular physiology in the 19th century, to the understandings of kidney function measurement and kidney disease origins.^1^ Despite the expansion of relevant knowledge base, therapeutic approaches capable of retarding kidney disease progression remain quite limited; only renin-angiontensin system inhibitors (RASi) demonstrated impressive outcome-improving efficacy in randomized controlled trials (1, 2) two decades ago. Nowadays, encouraging advancements have been made in the nephrology field. We would like to briefly summarize these inspiring changes and provide personal accounts on important and emerging topics deserving particular attention in the future (Figure 1).

**Figure 1:**
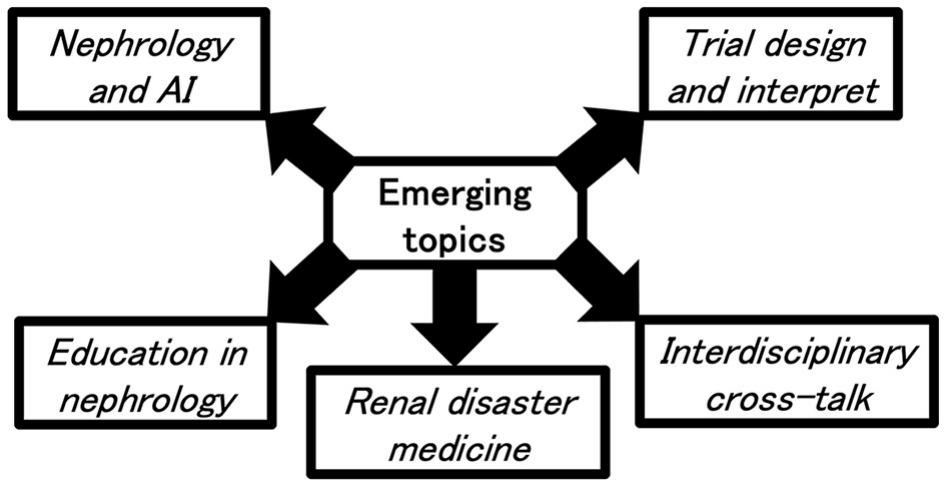
Emerging nephrology topics addressed in this opinion pieces.

## Impactful clinical trials and outcome diversification

We witness a rapid deployment of novel compounds for combating kidney diseases since 2015. Sodium-glucose cotransporter 2 inhibitors (SGLT2i) is among the first of its kind to exhibit renoprotective effects independent of its glycemic control capacity, as a class effect (3, 4). Subsequently, glucagon-like peptide-1 receptor agonists (GLP-1RA) (5) and newer generations of non-steroidal mineralocorticoid receptor antagonists (nsMRAs) (6) are also found to exhibit benefits in reducing the risk of composite renal outcomes in patients with chronic kidney disease (CKD). Through reducing glomerular hyperfiltration and intraglomerular pressure, optimizing afferent/efferent arteriole hemodynamics, conferring glomerular and tubular cellular metabolic benefits, attenuating kidney tissue hypoxia, these compounds pave the way toward engineering and testing the next generation of renoprotective agents (7). Aside from drugs reducing adverse kidney outcomes, there are also drugs approved for targeting immunopathology capable of managing distinct glomerulonephritis recently. Targeted release formulation of budesonide and sparsentan, an endothelin and angiotensin 2 type 1 receptor antagonist, have been shown to achieve proteinuria reduction among patients with IgA nephropathy (IgAN) (8). Small molecular and RNA-based complement inhibitors are promising candidates for ameliorating IgAN disease severities (9). These results lend support to the flourishing nature of kidney disease treatment regimens. Moreover, we can expect rising numbers of clinical trials evaluating nephrology treatments in the upcoming era. Nephrologists should familiarize themselves with the work of trialists and how to interpret trial findings, so as to select appropriate therapies for their patients. In addition, patient reported outcome measures (PROMs) gain momentum as key outcomes nephrologists should not overlook. Patients with kidney diseases may have significantly lower chance of PROM improvement compared to those without (10). Interventions aiming to increase ones' survival and chances of kidney preservation should not come at the price of worsening their symptom burden. We, at the meantime, look forward to accumulating evidence supporting softer outcomes of novel kidney disease treatments in the foreseeable future.

## Crosstalk between nephrology and other disciplines

Another important field would be the intersections between nephrology and other scientific disciplines. A well-established example is geriatric nephrology, addressing the fundamentals of senescence, how an organism ages, and how to care for older adults. Existing studies have pinpointed the rising prevalence and incidence of geriatric syndromes in patients with CKD, including frailty, sarcopenia, cognitive dysfunction, and disability. Among these degenerative phenotypes, frailty deserves particular attention, as its pathogenesis intertwines with that of CKD and its adverse influences extend far beyond survival (11, 12). Identifying frailty followed by administering dedicated managements carries the potential of improving CKD patients' functional status and quality of life. Ameliorating uremic sarcopenia and malnutrition is also feasible through appropriate dietary counseling and exercise regimen prescription. Newer fields spawning from inter-disciplinary collaboration further include onconephrology, nephrocardiology, and nephro-palliative care (13). Nephrology as a discipline, builds on the development of physiology, pathology, and anatomy, but consolidates itself when maintenance dialysis is created and enters clinical practice. Thus, nephrology originates from the piecework of century-old human knowledge, grows incrementally and forms a firm scientific discipline, and now evolves further “into complexity” (14), partly assisted by the omic technology. Nephrology has now been transformed into a discipline that welcomes and actively encourages intimate collaborations with other scientific and medical fields.

## Artificial intelligence (AI) applications in nephrology

AI significantly revolutionizes the modern medicine, as it provides an automated way to perform complex tasks with further enhancement enabled by self-learning from continuous data input. Nephrology researchers have been apt to incorporate AI into various aspects of this field. For instance, the Nephrotic Syndrome Study Network (NEPTUNE) group has already leveraged multi-layered analyses for kidney pathology classification and kidney disease progression risk prediction (15). The applications of AI have also been extended to evaluate and manage different diseases relevant to nephrology, such as gauging fluid balance among patients undergoing continuous renal replacement therapy (CRRT) (16), outcome prediction in peritoneal dialysis patients and kidney transplant recipients (17, 18). The ability of AI to handle complex, multidimensional data makes it particularly suitable for nephrology, a discipline with diverse influential factors interacting with each other. Other topics of nephrology in which AI has been deployed involve the optimization and individualization of dialysis prescription; healthcare records, wearable devices, and physiologic signals produced during dialysis sessions are utilized to estimate the probability of untoward medical event (19). Image reading in CKD-mineral bone disorder has also been automatized and accelerated with the assistance of AI technology (20). These topics vividly illustrate the speed of AI uptake as a tool in the research and clinical field of nephrology.

## Education in nephrology

Nephrology workforce has been dwindling in multiple countries around the world, and how to increase different background trainees' interest in this discipline warrants serious attention. The search for a better education context, modality or route, and content to enhance learning efficacy assumes importance. Trainees may feel ill-prepared for the contemporary nephrology practice, especially when they are at the forefront of facing technology enabled practice innovation (21). How to uphold the value of nephrology specialty for to-be nephrologists and junior ones remains under-explored (22). This will require individualization of education approach throughout the course of training and potentially be accomplished through careful mentoring. However, literature in this topic is scarce and urgently needed.

Patients with CKD also have increasingly complex care needs, but exciting treatments were previously in lack. We now have new and further upcoming novel therapeutic choices for retarding CKD progression, but we need to intensify the spread of relevant knowledge through continuous professional development. High quality nephrology care for exceptionally complex patients at different settings cannot be achieved without successful teams led by well-educated nephrologists. Professional education focusing on fostering the continuous growth of nephrologists should take into account important education theories and preferences, as shown in prior examples (23). Let's not forget that nephrologists are frequently in work-life imbalances, as they are overwhelmed by the mounting clinical workload. We expect for more emphasis on studies designing creative strategies related to all stages of nephrology education from trainees to established specialists, and also team members. This will help the community build a stronger backbone and be resilient to more challenges ahead.

## Disaster medicine in nephrology

Recently we have seen more and more natural and manmade disasters complicating the appropriate provision of nephrology care to affected patients worldwide. However, the establishment of timely response procedures is rarely emphasized or achieved. Earthquakes in South Korea, Japan, and Taiwan highlight the need for disaster preparedness in nephrology, particularly for hemodialysis units. Prior experiences suggested that effective disaster responses require both logistics and training (24). These responses include establishing clear protocols, maintaining emergency supplies, training staff in emergency procedures, and educating patients on disaster preparedness. Moreover, a coordinated response system involving local dialysis units, regional centers, and governmental agencies is crucial (24). The need for an enhanced preparedness is particularly important for regions with a high population density and a large number of dialysis population, such as Taiwan and South Korea. The potential impact of disasters on the provision of nephrology care is concerning, making robust disaster response plans more critical. Through implementing comprehensive preparedness measures, nephrology providers in high-risk regions can significantly improve their capacity to maintain continuity of care for vulnerable dialysis patients during disasters, saving lives and minimizing complications under challenging circumstances. The experience with COVID-19 underscores the need for nephrology care providers to develop and maintain specific protocols for managing infectious disease outbreaks (25). Such protocols may include measures for patient isolation, staff protection, continuity of dialysis services and proper vaccination (26).

## Conclusion

Nephrology is experiencing renaissance, with exciting developments across multiple aspects. The presence of impactful clinical trials testing novel therapies, increasing opportunities for interdisciplinary collaboration, and the integration of artificial intelligence exemplify this promising trend. These advancements reshape our existing approach of nephrology care and how we conduct nephrology researches. Simultaneously, this discipline faces challenges in education and workforce development, necessitating innovative strategies to attract and retain talented members. The emerging focus on disaster medicine in nephrology, particularly in regions with high dialysis populations such as South Korea and Taiwan, highlights the need for robust preparedness plans. It is therefore crucial for practitioners to stay abreast of these developments and contribute to the growth of future nephrology. We warmly welcome potential candidates to submit relevant and impactful manuscript to this section for consideration.

## Author contributions

KY: Conceptualization, Formal analysis, Funding acquisition, Investigation, Validation, Writing – original draft, Writing – review & editing. C-TC: Conceptualization, Data curation, Formal analysis, Funding acquisition, Investigation, Methodology, Project administration, Resources, Supervision, Validation, Visualization, Writing – original draft, Writing – review & editing.

## Footnotes

^1^ https://www.theisn.org/about-isn/history/breakthrough-discoveries/

## References

[1] Brenner BM Cooper ME de Zeeuw D Keane WF Mitch WE Parving HH. Effects of losartan on renal and cardiovascular outcomes in patients with type 2 diabetes and nephropathy. N Engl J Med. (2001) 345:861–9. 10.1056/NEJMoa011161 11565518

[2] Lewis EJ Hunsicker LG Clarke WR Berl T Pohl MA Lewis JB. Renoprotective effect of the angitensin-receptor antagonist irbesartan in patients with nephropathy due to type 2 diabetes. N Engl J Med. (2001) 345:851–60. 10.1056/NEJMoa011303 11565517

[3] Heerspink HJ Stefansson BV Correa-Rotter R Chertow GM Greene T Hou FF. Dapagliflozin in patients with chronic kidney disease with chronic kidney disease. N Engl J Med. (2020) 383:1436–46. 10.1056/NEJMoa2024816 32970396

[4] The EMPA-KIDNEY Collaborative Group Herrington WG Staplin N Wanner C Green JB Hauske SJ. Empagliflozin in patients with chronic kidney disease. N Engl J Med. (2023) 388:117–27. 10.1056/NEJMoa2204233 36331190 PMC7614055

[5] Li X Song Y Guo T Xiao G Li Q. Effect of glucagon-like peptide 1 receptor agonists on the renal protection in patients with diabetes mellitus: a systematic review and meta-analysis. Diabetes Metab. (2022) 48:101366. 10.1016/j.diabet.2022.101366 35760374

[6] Barrera-Chimal J Lima-Posada I Bakris GL Jaisser F. Mineralocorticoid receptor and antagonists in diabetic kidney disease – mechanistic and therapeutic effects. Nat Rev Nephrol. (2022) 18:56–70. 10.1038/s41581-021-00490-8 34675379

[7] Sen T Heerspink HJL. A kidney perspective on the mechanism of action of sodium glucose co-transported 2 inhibitors. Cell Metab. (2021) 33:732–9. 10.1016/j.cmet.2021.02.016 33691091

[8] Selvaskandan H Barratt J Cheung CK. Novel treatment paradigms: primary IgA nephropathy. Kidney Int Rep. (2023) 9:203–13. 10.1016/j.ekir.2023.11.026 38344739 PMC10851020

[9] Barratt J Liew A Yeo SC Fernström A Barbour SJ Sperati CJ. Phase 2 trial of cemdisiran in adult patients with IgA nephropathy: a randomized controlled trial. Clin J Am Soc Nephrol. (2024) 19:452–62. 10.2215/CJN.0000000000000384 38214599 PMC11020434

[10] Chao C-T Yang R-S Hung L-W Tsai K-S Peng J-K Chang C-H. Chronic kidney disease predicts a lower probability of improvement in patient-reported experience measures among patients with fractures: a prospective multicenter cohort study. Arch Osteoporos. (2018) 13:126. 10.1007/s11657-018-0539-0 30446836

[11] Wu PY Chao CT Chan DC Huang JW Hung KY. Contributors, risk associates, and complications of frailty in patients with chronic kidney disease: a scoping review. Ther Adv Chronic Dis. (2019) 10:2040622319880382. 10.1177/2040622319880382 31632625 PMC6778996

[12] Chan GC Kalantar-Zadeh K Ng JK Tian N Burns A Chow KM. Frailty in patients on dialysis. Kidney Int. (2024) 106:35–49. 10.1016/j.kint.2024.02.026 38705274

[13] Hatamizadeh P. Introducing nephrocardiology. Clin J Am Soc Nephrol. (2022) 17:311–3. 10.2215/CJN.10940821 34893503 PMC8823943

[14] De Santo NG. Nephrology a disciplinme evolving into complexity: between complex systems and philosophy. J Nephrol. (2020) 33:1–4. 10.1007/s40620-019-00674-3 31776945

[15] Gadegbeku CA Gipson DS Holzman LB Ojo AO Song PXK Barisoni L. Design of the Nephrotic Syndrome Study Network (NEPTUNE) to evaluate primary glomerular nephropathy by a multi-disciplinary approach. Kidney Int. (2013) 83:749–56. 10.1038/ki.2012.428 23325076 PMC3612359

[16] Yoo KD Noh J Bae W An JN Oh HJ Rhee H. Predicting outcomes of continuous renal replacement therapy using body composition monitoring: a deep-learning approach. Sci Rep. (2023) 13:4605. 10.1038/s41598-023-30074-4 36944678 PMC10030803

[17] Noh J Yoo KD Bae W Lee JS Kim K Cho J-H. Prediction of the mortality risk in peritoneal dialysis patients using machine learning models: a nation-wide prospective cohort in Korea. Sci Rep. (2020) 10:7470. 10.1038/s41598-020-64184-0 32366838 PMC7198502

[18] Yoo KD Noh J Lee H Kim DK Lim CS Kim YH. A machine learning approach using survival statistics to predict graft survival in kidney transplant recipients: a multicenter cohort study. Sci Rep. (2017) 7:8904. 10.1038/s41598-017-08008-8 28827646 PMC5567098

[19] Scherer L Kuss M Nahm W. Review of artificial intelligence-based signal processing in dialysis: challenges for machine-embeded and complementary applications. Adv Kidney Dis Health. (2023) 30:40–6. 10.1053/j.akdh.2022.11.002 36723281

[20] Chao CT Yeh HY Hung KY. Chest radiography deep radiomics-enabled aortic arch calcification interpretation across different populations. iScience. (2023) 26:106429. 10.1016/j.isci.2023.106429 37009230 PMC10050631

[21] Chao CT Hung KY. Emphasizing probabilistic reasoning education: helping nephrology trainees to cope with uncertainty in the era of AI-assisted clinical practice. Nephrology. (2024) 29:169–71. 10.1111/nep.14263 38109797

[22] Rosenberg ME Anderson S Farouk SS Gibson KL Jr RSH Humphreys BD. Reimaging nephrology fellowship education to meet the future needs of nephrology: a report of the American Society of Nephrology task force on the future of nephrology. Clin J Am Soc Nephrol. (2023) 18:816–25. 10.2215/CJN.0000000000000133 36848491 PMC10278777

[23] Chao C-T Wu M-Y Hung K-Y Wu M-S Liang J-C. Interprofessional differences in multidimensional self-efficacy associated with professional performance in nephrology during case-based learning. Kidney Int Rep. (2024) 9:877–87. 10.1016/j.ekir.2024.01.018 38765585 PMC11101767

[24] Yoo KD Kim HJ Kim Y Park JY Shin SJ Han SH. Disaster preparedness for earthquakes in hemodialysis units in Gyeongju and Pohang, South Korea. Kidney Res Clin Pract. (2019) 38:15–24. 10.23876/j.krcp.18.0058 30776874 PMC6481979

[25] Cho A Jeong SA Park HC Kim DH Yoo KD Yoon HE. Comparative analysis of the incidence and mortality of COVID-19 in Korean end-stage kidney disease patients: hemodialysis, peritoneal dialysis, and transplantation. Kidney Res Clin Pract. (2024). 10.23876/j.krcp.23.287. [Epub ahead of print]. 38934044 PMC12066348

[26] Lee YK Jeong SA Park HC Kim DH Yoo KD Yoon HE. SARS-CoV-2 vaccine effectiveness and clinical outcomes in hemodialysis patients: the NHIS-COVID-19 cohort study in South Korea. Front Public Health. (2024) 12:1372525. 10.3389/fpubh.2024.1372525 38784571 PMC11111925

